# Physical Activity on Prescription “Not a Quick Fix”: School Nurses’ Experiences of Promoting and Tailoring Physical Activity to Children in Swedish Compulsory School

**DOI:** 10.1177/10598405231166124

**Published:** 2023-03-27

**Authors:** Emelie Wiklund, Maria Wiklund, Susanna Hedenborg

**Affiliations:** 1 Department of Sports Sciences, Malmö University, Malmö, Sweden; 2 Physiotherapy Unit, Department of Community Medicine and Rehabilitation, 8075 Umeå University, Umeå, Sweden

**Keywords:** physical activity, physical activity on prescription, qualitative interviews, school nurses

## Abstract

This study aimed to explore school nurses’ experience of using physical activity on prescription with children in Swedish compulsory school. Semistructured interviews were conducted with 24 school nurses who had the educational qualification to prescribe physical activity. The analysis resulted in one overarching theme, “A delicate process of tailoring physical activity on prescription to a child's social context,” and two categories: “Promoting joyful physical activity through individualization and support” and “Dealing with dilemmas and challenges,” with related subcategories. The results demonstrate the importance of tailoring prescribed physical activity to each individual child, their living conditions, and the school context. In addition, they highlight the nurses’ working conditions and collaborations as important prerequisites when initiating physical activity on prescription. In conclusion, the results suggest that school nurses perceive physical activity on prescription as a useful tool in the school context, but it needs to be tailored to each individual child.

## Introduction

Physical activity (PA) is crucial for children's health and well-being. PA reduces the risk of diseases such as type 2 diabetes and cardiovascular disease and promotes children's learning and concentration skills and their mental health (Chaput et al., 2020). Children are generally recommended to engage in moderate to vigorous PA for at least 60 min per day and to limit their sedentary time (Chaput et al., 2020). Globally, only 19% of children aged 11–17 years reach this recommendation (Guthold et al., 2020). In Sweden, 43% of boys and 23% of girls aged 11–18 years reach this recommendation (Nyberg et al., 2020). Research indicates that children and adolescents have different prerequisites to reach the recommendations (World Health Organization, 2021). Socioeconomic vulnerability (Elgar et al., 2015; Rosell et al., 2021; WHO, 2016), female gender (Guthold et al., 2020), and disability (Carty et al., 2021) are highlighted as particular barriers. The consequences of this are increased health gaps, which are costly for both the individual and the society (WHO, 2018). Reducing inequality in health is highlighted in the 2030 Sustainable Development Goals (United Nations, 2015), and equal health for children and adolescents is also a right according to the United Nations Convention on the Rights of the Child (1989). Thus, in a society where the population is becoming increasingly sedentary (Guthold et al., 2020) and the health gaps are increasing (WHO, 2018), it is important to create conditions for all children and adolescents to be physically active.

Physical activity on prescription (PAP) is an individually written recommendation of PA (Public Health Agency of Sweden, 2011). Studies with adults show that PAP increases the level of PA and decreases sedentary time (Kallings et al., 2008; Onerup et al., 2019). PAP can be used by school nurses to support children who do not reach the health recommendation for PA and who are at risk of illness (Public Health Agency of Sweden, 2011). The prescription has primarily been used and evaluated in healthcare (Boman et al., 2023; Lauruschkus et al., 2017; Lydell et al., 2022; Thorén et al., 2021) and is still unexplored in the school context.

School is an important and cost-effective health promotion arena with the potential to reach all children (Kriemler et al., 2011; Sheehan et al., 2017). School health services (SHS) are key in health promotion (WHO, 2019) and in Sweden, SHS are regulated by the Education Act (The Education Act., SFS 2010:800). All children and adolescents are legally entitled to SHS, which are free of charge for all children regardless of socioeconomic position. Their mission is to promote health, disease prevention, and remedial action—including medical, psychological, psychosocial, and special educational interventions (The Education Act., SFS 2010:800). In SHS, the school nurse has a central role together with other professionals.

In summary, studies indicate that school-based health promotion activities can be effective (Milton et al., 2021), and there is evidence that PAP is an appropriate method to increase PA in adults (Kallings et al., 2008; Onerup et al., 2019). However, evidence for PAP as an appropriate tool for children in the school context is lacking. Since school nurses provide PAP in school, they have an important role in evaluating PAP's effectiveness and implementation. Therefore, this study aimed to explore school nurses’ experience of using PAP with children in Swedish compulsory school.

## Method

### Study Context

The study was conducted with school nurses from SHS using PAP in compulsory schools in southern and central Sweden. Swedish SHS includes school nurses, a school doctor, a psychologist, and a counselor, who offer medical, psychological, psychosocial, and special educational interventions. The primary role of SHS is disease prevention and health promotion. In Sweden, SHS are regulated by the Swedish Education Act (The Education Act., SFS 2010:800).

### (Co-Organized) Physical Activity on Prescription

PAP is an evidence-based method initially developed for health care providers to support people in increasing their PA (Public Health Agency of Sweden, 2011). All licensed Swedish healthcare professionals with adequate expertise have the mandate to prescribe PAP, including school nurses in compulsory school (Public Health Agency of Sweden, 2011). PAP activities can be implemented into the school day (e.g., through specified PA time during recess and outside of school time (most common in this study). The prescription can include individualized PA, for example, everyday exercise or organized PAP activities led by an activity organizer, but it can also involve subsidized activities, such as swimming and gym workout. PAP is based on a person-centered approach and is organized according to a template with three steps: person-centered dialogue, written prescription for PA, and individualized follow-up (Public Health Agency of Sweden, 2011). The person-centered approach implies variation regarding the type of PA and its duration. The school nurses in this study had previous standardized education on how to conduct the three steps of PAP. The initial PAP dialogue with the child was usually carried out during a regular health visit, offered to each child at least three times during their total school time. Children who did not reach the health recommendations for PA and were at risk of illness were offered PAP. The most common reason for prescribing PA was obesity.

In this study, some of the participating school nurses in certain municipalities had a PAP supplement: Co-organized PAP. Co-organized PAP involves increased collaboration around the PAP model and means that the children have a supportive environment in the form of a network of adults. The child chooses a support person, for example, school staff or a parent (in this article, the term “parent” also includes legal guardians of children), whose mission is to stimulate the child's motivation by supporting, encouraging, and paying attention to the child during the prescription period.

### Participants and Procedure

School nurses were strategically selected to get a variety of schools, sociodemographic conditions, and experiences in using PAP. They were recruited from 24 SHS in compulsory schools (for children 6–16 years old), located in six municipalities with a differing number of inhabitants (small, medium, and large cities) and with varied geographic areas (rural and urban). According to the Swedish National Agency for Education (2020), the selected schools’ socioeconomic indexes ranged from 33.7 to 289.2 (a higher number equals higher socioeconomic vulnerability). Several schools were located in areas with a high proportion of residents with a foreign background, particularly from non-EU countries. Some schools were located in areas ranked as Sweden's most vulnerable, characterized by parallel social structures: low socioeconomic status, low income, and low educational level. There was a wide variation in the children's first language (Arabic and Somali were common) and the parents’ country of birth.

Initially, contact was made via email with key persons in SHS, with information about the study and the researcher attached. Thereafter, all school nurses in the selected municipalities were contacted via email, and 24 of 200 school nurses expressed an interest in participating. Thus, the study included 23 women and 1 man (a representative sample); they had 1–20 years’ (7.5-year mean) experience of working in SHS and 0.5–7 years (2.5-year mean) of using PAP. All participants gave oral informed consent to participate in the study. Ethical approval was obtained from the Swedish Ethical Review Authority.

The participating school nurses worked with different age groups in compulsory schools: 13 nurses with the age group 6–12 years, 4 nurses with the age group 13–16 years, and 7 nurses with the age group 6–16 years. The recruitment and interviews with the participants continued until theoretical saturation was reached. After the 24 interviews, the researchers considered theoretical saturation was reached.

### Data Collection and Analysis

The data were collected through individual interviews with 24 participants. The interviews were open-ended and semistructured (Graneheim & Lundman, 2004). with an interview guide exploring experiences related to five thematic areas (Table 1). Before the interviews, one pilot interview was conducted to fine-tune the interview guide. The interviews were conducted by the first author either by videoconferencing tools (Zoom or Teams) or by mobile phone, and they were recorded. The interviews lasted between 36 and 57 min (43-min mean).

**Table 1.** Questions in the Interview Guide.

Background information • The school nurse's number of professional years in SHS • The school's socioeconomic index • Description of the children's physical status at school
Thematic areas • SHS function and competence • Prescribing of PA (barriers and facilitators for the use of PAP) • Collaborations • Prospects
Each interview was supplemented with follow-up questions based on the participants’ answers about experiences of using PAP.

The study used qualitative content analysis with an inductive approach (Graneheim & Lundman, 2004). First, the verbatim transcripts of each interview were summarized to capture the “naive” understanding and get a sense of the whole. Thereafter, meaning units were condensed and provided with codes, which interpreted the content on a slightly higher level of abstraction (while remaining closely related to descriptive data), supported by the software NVivo. Codes with similar meanings were then brought together into subcategories, forming two categories. Finally, one main theme was formulated that captured the overarching and latent content running through the categories (Graneheim & Lundman, 2004). As a part of triangulation, used to strengthen reliability (Graneheim & Lundman, 2004), interpretations throughout the analysis were continuously discussed between all three authors, who have different competencies and perspectives (e.g., sports science, child and adolescent science studies, public health, and physiotherapy). Any disagreements on the content of themes were resolved through group discussion with all authors. After discussions between all authors, consensus was reached.

## Results: A Delicate Process of Tailoring PAP to a Child's Social Context

The results revealed an overarching theme: “A delicate process of tailoring PAP to a child social context.” It reflects the school nurses’ discrepant and sometimes ambivalent experiences of navigating PAP and their specific expertise and receptiveness in doing so. Their different experiences are divided into two categories: “Promoting joyful physical activity through individualization and support” and “Dealing with dilemmas and challenges,” (with related subcategories detailed in Table 2).

**Table 2.** Subcategories, Categories, and Themes.

Subcategories	Categories	Theme
• School as a safe environment for health promotion • PAP as a motivation booster and joy creator for the whole family • Adaption to the child • A large area of use • The importance of a supportive network • Collaboration important to consider the whole concept of health	Promoting joyful physical activity through individualization and support	A delicate process of tailoring PAP to a child's social context
• Ethical dilemmas with the (unequal) range and access of activities • Balancing between preventing illness and stigmatization • The important but hard cooperation with guardians • A time-consuming process • More resources are needed to work with PAP	Dealing with dilemmas and challenges	

### Promoting Joyful PA Through Individualization and Support

The school nurses highlighted the possibilities and positive experiences of PAP, particularly when individualized to each child (see Table 2). The school nurses who primarily saw the benefits of PAP worked in a context that facilitated the process by allowing a well-functioning collaboration with the school and families and giving the school nurses dedicated time and authority to work with PAP. A school with such circumstances was considered a suitable arena for PAP because it is an “environment in which many children and parents feel safe” (Interview 20).

To promote PA, the school nurses had close dialogue and explored what the children thought was fun. This gave the school nurses the potential to create “joy-filled” PAP and PA. They underlined the importance of not only telling the health benefits of PAP but also exploring intrinsic motivation.

It's about finding the motivation and the joy. What is it that the child likes? That's where you have to start. And capture… and create change in the long term. It has to be joyful and motivating. Because that's our mission. To create joyful change. (Interview 10)

In general, the school nurses said PAP had the potential to promote healthy PA in schoolchildren in addition to other benefits, such as social support. PAP was described as a “ motivation booster” for PA, creating joy in body movement. According to the school nurses, children who were not previously physically active benefitted from PAP:

We give the child attention. Often, it's children who have not felt so good before. And suddenly we have something positive to talk about. (Interview 16)

The positive benefits of PAP were seen not only in the children but also in the whole family:

It's a great way to try to motivate parents to take their children to activities. I think it is fantastic that it exists and that you can try to guide the families. (Interview 3)

According to the school nurses, the key to success in using PAP in a school context was individualizing PAP and relating it to the child's social context. Children were considered to have different prerequisites for PA that could be met by PAP.

The nurses perceived PAP as having broad areas of application, such as strengthening physical and mental well-being. Another stated advantage was the “status in the prescription heading” (Interview 5), which helped strengthen the importance of and interest in PAP among the families.

The school nurses expressed that they often felt alone in their role at school, but PAP led to collaboration with other professionals, which made them feel more secure. School nurses with access to a local support network for the child (co-organized PAP) said the collaboration had other positive outcomes:

I think it gives rings on the water, and the project with PAP has led to the fact that you can also do other activities [at school], which have to do with PA. It creates an awareness. (Interview 16)

Further, the school nurses found it important to not only focus on PA but also problematize and approach “the whole concept of health.” Therefore, collaborating with authorities, municipalities, schools, and families was a crucial precondition.

### Dealing with Dilemmas and Challenges

When prescribing PA, the school nurses experienced dilemmas and challenges, primarily related to external circumstances impossible to control by the school (see Table 2). The unequal range and accessibility of activities, which varied between municipalities, was an example. In addition, the school nurses perceived that the location of the activities was a challenge because they were mainly offered in city centers, which made it difficult for children from rural schools and suburbs with a socioeconomically vulnerable population to gain access. Some school nurses prescribed PAP in the form of everyday exercise, which they described as difficult in areas with overcrowding and high crime rates. They were afraid that the children could not perform PA either at home or outside in the immediate area. Another challenge put forward was costs. The PAP activities were free in some areas and required fees in others. This gave rise to ethical dilemmas, too:

I don’t want it [PAP] to be stigmatizing in any way. I want everyone to be physically active, but it cannot be the case that “yes, but your parents can buy or send you to exercise.” But then someone else says you can’t afford it. I think it will be a difficult situation. Or some ethical dilemmas. (Interview 17)

Some school nurses described it as a “balancing act”—talking about PA and health habits while simultaneously not blaming or stigmatizing the children and parents who, for example, are overweight. One group that offered PAP was children with obesity. The school nurses felt that PAP was a concrete tool that helped them balance preventing illness with PA and avoiding stigmatizing children with obesity.

The school nurses saw cooperating with parents as a requisite for successfully using PAP. However, they identified some groups as more difficult to reach, for example, parents who did not understand the Swedish language were not committed or motivated to support their child, or did not have the ability or financial means. School nurses active in multicultural areas pointed out differences in perception of PAP, forming a challenge due to a potential “clash” between the residents’ view of PA and school nurses’ work with PAP.

I think there are cultural things as well. In some countries, children are not to be physically active before they have grown up. The child can be very sedentary. So, there are probably different aspects to it. And ignorance too. (Interview 3)

To counteract the effect of segregation, the school nurses told how they invested in giving information and having close contact with families. The collaboration was seen as a difficult but important prerequisite for success with PAP.

It is very difficult with collaboration, but sometimes you succeed. But it is a delicate work with both children and parents. (Interview 7)

However, collaboration was perceived as a demanding process that was not always possible, depending on the school nurse's own working conditions. A shared experience was that using PAP was time-consuming. It was not a “quick fix” but a demanding and “ delicate” process in which all conditions must be considered. Some school nurses had previously prescribed PAP but now “opted out” of the tool due to lack of time.

Furthermore, the collaboration with educators and physical education teachers was viewed as negatively affected by lack of time. Consequently, the school nurses requested more resources and professionals who work specifically with PA in the school context:

We have talked about that several times. To have a physiotherapist at school who can work with the children. (Interview 6)

According to the school nurses, collaboration and more allocated time gave them relief and support, which they thought resulted in more children benefitting from PAP.

## Discussion

This study investigated school nurses’ experiences with PAP. The results demonstrate that they perceive PAP as a useful tool for joyful health promotion and motivation, but it is not to be seen as “quick fix”: It is a complex, challenging process where considering the child's social context is key.

The results suggest that prescribing PA to children in a school context is a more complex and “delicate” process than previous research with adults has shown (Kallings et al., 2008; Onerup et al., 2019). Although the school is a health-promotion area with the potential to reach all children (Kriemler et al., 2011), the school nurses’ experiences indicate that social aspects—such as the school's socioeconomic index and location and the possibility of collaborations with parents and school staff in segregated and multicultural areas—influence whether PAP is a successful tool in this context.

Contextualizing and tailoring’ proved to be crucial strategies for utilizing and developing PAP in a new environment (school) and for a new target group (children). This result is important for future PAP initiatives and complies with the person-centered approach (Kime et al., 2018) while remaining context-sensitive (Verjans-Janssen et al., 2020). Furthermore, school nurses’ tailoring PA to the children's needs and the school context has previously been successful in obesity intervention (Schroeder & Smaldone, 2017). Likewise, school nurses’ application of a person-centered approach has proven valuable for children with recurrent pain (Wigert et al., 2021). Our study contributes to these perspectives on promoting PA by using PAP in a school context.

The school nurses described PAP as a balancing act: They want to prevent illness with PA but not stigmatize the children who need PA (e.g., children with obesity). Previous research emphasizes the ethical and moral problems with approaching the “obesity epidemic,” in which overweight children risk being stigmatized (Gard & Wright, 2005). Gard and Wright (2005) argue for the need for biopedagogical practices that address social and societal factors, in contrast to hegemonic pathogenic health discourses that classify individuals as “good” or “ bad” citizens. Like the school nurses, Gard and Wright (2005) describe health promotion as a “balancing act,” where health risks of physical inactivity and obesity need to be balanced against the stigmatization of overweight people (which may lead to mental illness).

An important result revealed by the school nurses was that there was status in the prescription label that helped strengthen interest in PA and PAP among the children and parents. Given that PAP does not include a medicine prescription, it symbolizes health and well-being rather than illness. Therefore, PAP can potentially be a positive complement to, for example, the increased (pill-form) medicalization of children and adolescents (National Board of Health and Welfare, 2019). The results also raise crucial questions about the ethical aspects of prescribing PAP to children. Namely, when the procedure of writing a “prescription,” originating from a medical context, is transferred to a school context, there is a risk of unnecessary medicalization (Public Health Agency of Sweden, 2011).

The results show how the school nurses navigated certain aspects of using PAP, mainly in relation to the school context and the children's discrepant living conditions. The school nurses considered some children “ hard to reach,” particularly in multicultural areas with low socioeconomic status, and found close collaboration with parents an important countermeasure. The collaboration was sometimes challenging, which has also been shown in previous research (Mäenpää et al., 2013). Likewise, Elgar et al. (2015) argue that health promotion should consider the social determinants of children's PA. Evidently, socioeconomic inequality is increasing and has become apparent in the health of children, and children with a low socioeconomic status are less physically active (Elgar et al., 2015; Rosell et al., 2021; WHO, 2016). To even out this inequity, the school nurses called for free PAP activities on school premises. The results are strengthened by previous research, which shows that school-based health promotion activities are positive (Milton et al., 2021).

Linked to the 2030 Sustainable Development Goals (United Nations, 2015) and the public health policy goals in Sweden (Public health agency of Sweden, 2022), a challenge for the school nurses was their working conditions, as implementing and tailoring PAP are time-consuming. Furthermore, the school nurses highlighted work-related dilemmas and challenges reflecting society at large. For them, PAP was not a “quick fix” that could solve every health issue but rather a tool that could yield results if given enough resources. This is important because, according to the Swedish law (The Education Act., SFS 2010:800), all children have the right to get support from the school nurse. Therefore, school nurses have an important mission to be available for all children and create equity in health for all children.

The school nurses considered PAP a useful tool with potential. PAP enabled them to explore children's inner motivation and individualize PA. By exploring inner motivation in children, school nurses could create joy in body movement. School-based PA interventions have been proven effective if there is intrinsic motivation (Kelso et al., 2020), and joy in body movement has been proven to strengthen the motivation to be more physically active (Duberg et al., 2016; Pearson et al., 2015). In congruence with the United Nations Convention on the Rights of the Child (1989), it is also important to allow children to be involved in deciding which PA they will perform. Physically inactive children, without a sports background, often request activities based on their own interests, such as playing, walking, or jumping on a trampoline (Högman, 2021). Such PA is possible via PAP and is in line with the school nurses’ thoughts about individualizing the PAP and facilitating the child's intrinsic motivation.

Moreover, using PAP increased collaboration between professionals, creating a network to support the children's PA. Social support, such as from parents and school staff, is a requisite for children's lifestyle changes and increased PA (Bonde Høstgaard et al., 2014; Khan et al., 2020; Mendonca et al., 2014; Moberg et al., 2021). Parents of children with obesity who received PAP and a web-based support program from the healthcare system find involving the school important to maintain a healthy lifestyle (Thorén et al., 2021). This is in line with this study's results, where compliance with PAP in a school context is dependent on the child's social context.

As knowledge about PAP for children is still lacking (Kallings et al., 2021), the present study is an important contribution. Future studies from various contexts can provide additional perspectives, including those concerning children.

### Strengths and Limitations

Regarding the study's method, the chosen qualitative and explorative approach was a strength (Graneheim & Lundman, 2004) as it captured school nurses’ experiences of daily work with PAP close to the target group, schoolchildren. Another strength is the broad variation in experiences created by the strategic sampling of school nurses from different schools, SHS, and municipalities with different socioeconomic conditions and ethnicities.

A possible limitation can be a selection bias. Namely, only school nurses with a specific interest in PA and PAP may have signed up for the study, whereas those with less interest in PAP might have been less inclined to participate. How many students the school nurse was responsible for might be of importance for the perceived work situation regarding the category “Dealing with dilemmas and challenges.” Therefore, it may be a limitation that information about this is missing in the article. Another limitation may be that the authors are not school nurses and have no experience of working in SHS; therefore, they may have missed something important in the SHS context. However, being “outsiders” may also be an advantage, and two of the authors have a professional background as physiotherapists and clinical experience in tailoring PA (first and second author) and prescribing PAP (first author), which gives an insider view of PA and PAP. The third author has researched children's PA in nonmedical contexts, providing perspectives outside of the “medical gaze.” Using triangulation between researchers with different backgrounds (Graneheim & Lundman, 2004) throughout the analysis increased the reliability.

The interviews were conducted remotely due to the COVID-19 pandemic, which had both pros and cons. The use of digital tools may have been unfamiliar to participants, and the lack of certain body language may have influenced dialogue and its interpretation. On the other hand, the digital format facilitated recruitment and participation (Archibald et al., 2019).

## Conclusions

The results demonstrate that school nurses’ use of PAP with school children is not a “quick fix” but a “motivation booster” and a joy creator. Using PAP in SHS is a complex and delicate process of tailoring to the needs and social conditions of each child. This delicate process needs both competence and organizational resources.

### Implications for School Nursing and Future Research

School nurses have a central role in SHS, and they can prescribe PAP to support children who do not reach the health recommendation for PA and are at risk of illness (Public Health Agency of Sweden, 2011). The results in this study demonstrate that PAP is a useful and concrete tool for school nurses to promote PA—if it is individualized to each child. The individualized prescription is a strength that enables the school nurse to prescribe PA based on the school's conditions and the child's preferences for PA and place. The results also show tailoring PAP to the needs and social conditions of each child is a delicate process. Therefore, school nurses who want to implement PAP in their regular work must be well prepared and aware of the need to adapt PAP to each individual child. Otherwise, there is a risk that their work with PAP will not succeed. They also require relevant conditions in the form of competence development and organizational resources. Future studies should investigate children's own experiences of PAP in a school context. They could focus on, for example, different age groups, as there could hypothetically be a variation in the perception of PAP depending on the child's age. There is also a need for research into parents’ and other school staff's experiences of PAP. This knowledge may help school nurses navigate the delivery of PAP to children in the school context.
